# Do submissions entitled to an auto-waiver take more time to be accepted by open access journals?

**DOI:** 10.1186/1756-0500-7-238

**Published:** 2014-04-15

**Authors:** Riaz Uddin

**Affiliations:** 1 Department of Pharmacy, Stamford University Bangladesh, 51-Siddeswari Road, Dhaka 1217, Bangladesh

**Keywords:** Article processing charge, OA publication, Publication ethics, Transparency

## Abstract

**Background:**

Open access initiative is a “talk of the town” in scientific community in recent years. Many open access publishers have an auto-waiver policy for resource limited countries. It is still not documented that whether submissions from auto- and non-waiver countries take the same time to be accepted by the editorial office or a sense of priority works for non-waiver groups.

**Findings:**

Analyzing 248 articles published in BMC Research Notes in 2013 we have found that average 143.8 ± 5.134 and 138.4 ± 12.01 days respectively for non-waiver and auto-waiver countries were required by the editorial office to accept a submission (*p* = 0.6983).

**Conclusion:**

From this current investigation it is quite evident that both categories of submissions, coming from auto- and non-waiver countries, are equally treated by the for-profit open access journals and thus it can be concluded that no sense of priority works in case of submissions those come from non-waiver countries.

## Findings

### Introduction

“Open access” has become a brand in previous few years in the academia, more specifically in journal publishing. The concept of OA is widely labeled as either “green” or “gold” open access. While the second one, gold open access, is considered by experts [1] as OA journals, the green open access refers to publishers those allow “self-archiving” of articles [2], typically by the author or a third party either in a subject repository; i.e. arXiv, or in a institutional repository. At the infancy of OA concept, around the year of 2003, most of the OA journals decided to make their articles available in the Web free of charge. At that time the authors did not need to pay for publishing, rather the journals founded by independent academics or already established societies took it as a responsibility which they did voluntarily [1]. However, this early model of OA journal seemed to be financially nonviable and thus “author-pay” OA model of academic publishing began its journey [1,3]. Nowadays several subscription based publishers are getting involved in OA publishing, adopting their own OA business model and the number of OA journals are growing every year.

The basic concept of author-pay OA publishing is the author, or the author’s institution on behalf of the author, will pay a “publication fee”, which is frequently termed as “article processing charge”, for an article to be published in an OA journal. In this process the article remains open, either freely downloadable as PDF document or available as HTML or in any other format so that the readers do not need to pay or subscribe to read the article.

Despite few negative criticisms [4,5] OA publication policy is well praised among the scientific community [6-8]. Especially the students, academicians, researchers and authors coming from low resource countries have been benefited by this policy as they need not to pay for reading an OA article. As this publication method requires the author to pay an article processing charge, it is hard for the researchers from low income countries or countries where resources are limited to publish their articles in these journals. But the good thing is most of the OA publishers with a good reputation adopted an auto waiver policy for the resource limited countries thus making it possible for the authors coming from such a country to enjoy OA publishing.

Peer reviewed journals follow a complex “author-editor-reviewer” interrelationship process. The process starts with the author submitting a manuscript to a journal, the editor initially assess the suitability of the submission and if suitable send it to peer reviewers following their own peer review policy. The reviewer submits his report and after assessing the pros and cons the editor takes the decision to publish or to reject the manuscript. So, the process demands coordination and synchronization among the author-reviewer-editor. There are many cut off points where a delay may occur and more time may be required for a particular submission to be accepted. The first hurdle in this process is to get a “go” card from the initial editorial processing. A delay in this point may result in a delayed acceptance of the manuscript. So, a sense of priority may work for the submissions coming from non-waiver countries and thus the editorial office may prioritize the submissions on the basis of capability of the author to pay the processing fee. And if there is, submissions from countries that get an auto waiver may take more time to be accepted by the editorial office over regular (non-waiver) submissions.

This article will test whether the OA publishers who offer a waiver on article processing charge treat all the submissions equally or not. However, it is worth mentioning that there are many non-profit OA journals, usually operated by educational institutions or societies; do not charge the authors any article processing fee. This article will deal only with Gold OA author pay journals.

## Methods

Our null hypothesis, H_O_ = author’s ability to pay has no effect on time required to accept a submission, and the alternative hypothesis H_1_ = submissions from auto-waiver countries take more time to be accepted than submissions from non-waiver countries. If we can establish that H_O_ is true we may prove that no sense of priority works in case of accepting a manuscript though the article processing charge is waived.

To prove our hypothesis we have considered first 250 articles published in 2013 in BMC Research Notes. We have selected this particular journal as (a) it is published by BioMed Central, one of the most popular well reputed OA publisher; (b) it is not a subject selective journal and publishes across all fields of biology and medicine; (c) encourages publishing short publications, scientifically sound, high quality but not necessarily novel in its field thus allowing submissions around the world both technologically advanced countries and resource limited countries; (d) offers auto waiver to low-income economies or lower-middle-income economies as of September 2012, and which have a 2011 gross domestic product of less than 200 billion US dollars [9].

We excluded two articles from the analysis; one by Schwarte et al. [10] as it has no date of submission and annual acknowledgement of manuscript reviewers by Aime [11]. Remaining 248 articles were checked for the author’s country, date of submission and date of acceptance. We will consider the time required to accept an article by the editorial office, not date of publication. As per BioMed Central auto-waiver policy, a submission is entitled to receive an auto-waiver if the corresponding author is based on, more specifically if the corresponding author's mailing address is located in a country covered by the publisher’s Open Access Waiver Fund. So, corresponding author’s email address was taken into consideration to identify the submissions those were supposed to receive an auto-waiver. For example, the article by Brouwer et al. [12] is authored by investigators from Mozambique and Netherlands, where the first country is covered by BioMed Central’s Open Access Waiver Fund and the other country falls in the non-waiver group. So, this article was considered as an auto-waiver one as the corresponding author is based in Mozambique.

The time span of a submission was calculated by subtracting the date of submission from the date of acceptance using Microsoft Excel. Two tailed non parametric T test was performed to confirm whether auto- and non-waiver groups differ in terms of total time required to accept the manuscripts and one way analysis of variance (ANOVA) was performed to observe any difference in average submission-to-acceptance time span for different type of articles using GraphPad Prism 5.0 for Windows. A *p* value less than 0.05 was considered to be statistically significant.

## Result and discussion

Among 248 articles being analyzed, 136 (54.84%) are research articles, 55 (22.18%) short reports, 81 (32.66%) technical notes, 45 (18.15%) case reports, 4 (1.61%) correspondences and 1 (0.40%) project note. ANOVA was performed for 247 articles excluding the project note as only one such article was found amongst the articles being analyzed. ANOVA confirms that type of articles is not associated with submission-to-acceptance time span (F = 1.003, *p* = 0.4033). Average time required to accept different type of articles is presented in Table 1.

**Table 1:** Average time required for different types of articles

**Type of article**	**Research article**	**Short report**	**Technical note**	**Case report**	**Correspondence**
**Minimum required time (day)**	1	4	15	27	70
**Maximum required time (day)**	498	314	209	282	207
**Average time required (day)**	148.5	150.7	122.3	133.6	124.3
**Standard deviation (day)**	82.89	71.23	43.38	69.14	64.23

**Figure 1:**
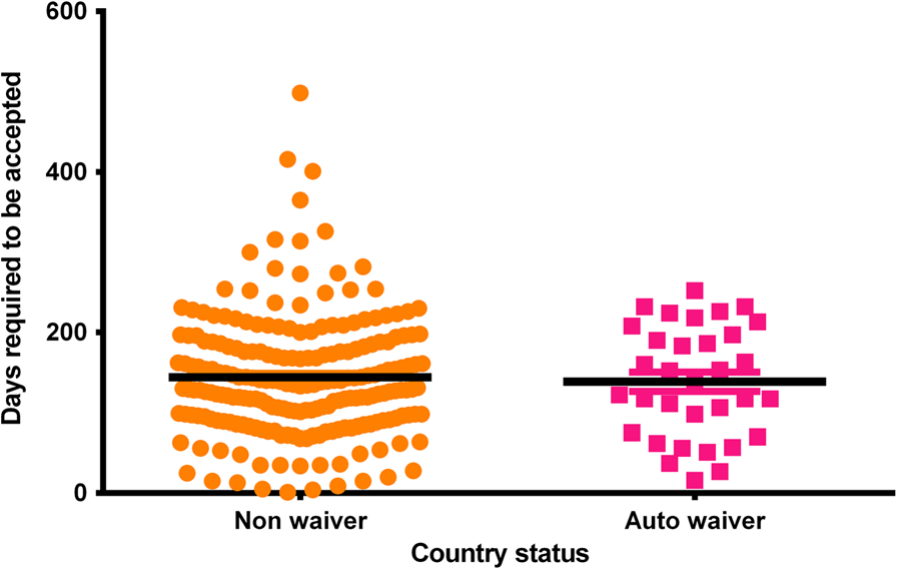
Time required for the articles to be accepted by the editorial office.

Among 248 articles the submissions from non-waiver countries took average 143.8 ± 5.134 days (Mean ± SEM) from date of submission to date of acceptance. Whereas, in case of countries entitled to have an auto-waiver the average time to be accepted by the editorial office was 138.4 ± 12.01 days. Figure 1 illustrates the distribution of time required for the articles to be accepted by the editorial office. With a *p* value of 0.6983, we can say that we have failed to reject the null hypothesis.

There are some extreme outliers in the non-waiver group. We have found three articles those took more than 365 days to be accepted. If we exclude these three articles from the analysis, no significant difference is observed in the results and in this case for non-waiver group the average time span for submission-to-acceptance is 139.6 ± 4.592 with a non significant difference for auto- and non-waiver groups (*p* = 0.9218).

It is statistically evident that there is no significant difference between auto- and non-waiver groups in terms of accepting a manuscript to be published in a for-profit OA journal. However, the total time required for a manuscript to be accepted is perhaps related to many other factors; i.e. finding potential peer reviewer(s), total time required to complete peer review by the reviewer(s), authors’ promptness to respond to the reviewers comments. Moreover, linguistic quality of the submission also might play an important part in the process. A manuscript written by a non-native English speaker might need additional work to improve quality of written English, thus extra time might be required for all parties concerned in the overall process. Furthermore, one can argue that type of article (research article, short report, case reports) may also play an important role in case of total required time. But as per the peer review policy of the publisher all type of articles undergo the same standard rigorous review process, regardless the type of article. Our analysis also confirms this as ANOVA reveals that article type is not statistically related to submission-to-acceptance time span (F = 1.003, *p* = 0.4033).

It is interesting to note that there are few articles which were immediately accepted upon submission. For instance, the article by Brorson et al. [13] required only one day to be accepted (submission date: 6 February 2013 and acceptance date: 7 February 2013). Without an editorial capacity in the journal it is not possible to explain how a manuscript can undergo all necessary editorial processing and required peer review process and subsequently be accepted within one day. However, it is possible to explain such a case by assuming that the manuscript was submitted in another BioMed Central journal and peer review of the manuscript was performed by that journal. Later on, it was probably transferred to BMC Research Notes and the editorial office immediately accepted the manuscript considering the suitability of the submission to be published in the journal and the previous peer reviewer reports deemed sufficient to accept the manuscript by them. However, this explanation seems to be the fact rather than an assumption as I received a personal comment from the Executive Editor, BMC Research Notes confirming that the manuscripts accepted within days of submission had all previously been reviewed in other BioMed Central journals and the editors of BMC Research Notes were able to accept them based on the previous review reports following transfer to the journal.

## Conclusion

So from this current investigation we can conclude that though the submissions by authors from auto-waiver countries enjoy a full waiver in many OA journals they are equally treated by the editorial office and no sense of priority works in case of non-waiver or auto-waiver countries. In other words we can say the financial part of the editorial process is sufficiently separated from the quality assessment process of the journal. However, every year if the publishers themselves publish this data it would make OA publishing more enjoyable, interactive and transparent.

## Competing interests

The author is a member of the editorial board of International Current Pharmaceutical Journal, an open access journal and received 30 USD for reviewing manuscripts for the journal. He is also a non paid Associate Editor of Stamford Journal of Pharmaceutical Sciences which is also open access.
